# Sustainable aquatic resource management and inland fisheries in tropical Asia: Interdisciplinary and transdisciplinary approaches

**DOI:** 10.1007/s13280-024-01996-8

**Published:** 2024-03-18

**Authors:** Fritz Schiemer, Upali S. Amarasinghe, David Simon, Jacobus Vijverberg

**Affiliations:** 1 Department of Functional and Evolutionary Ecology: Limnology, University of Vienna, Djerassiplatz 1., 1030 Wien, Austria; 2 Department of Zoology and Environmental Management, University of Kelaniya, Kelaniya, 11600 Sri Lanka; 3 Department of Geography, Royal Holloway, University of London, Egham, Surrey TW20 0EX UK; 4 Netherlands Institute of Ecology (NIOO-KNAW), Droevendaalsesteeg 10, 6708 PB Wageningen, The Netherlands

**Keywords:** Co-management, Food web interactions, Small indigenous species, Stakeholder participation, System approach, Transdisciplinarity

## Abstract

The intensive utilization of tropical inland water bodies for multiple and sometimes competing activities underlines the necessity for their integrated and holistic co-management. This paper presents our synthesis on lake and reservoir fisheries in South and Southeast Asia as social–ecological systems, based on a synopsis of our research findings from a previous EU-funded research programme in Sri Lanka, Thailand and the Philippines (FISHSTRAT project). The paper attempts to merge our results with recent developments in research, policy and practice. We explore the effects of the main external and internal control mechanisms of the trophic state and pinpoint to the high production potential of traditionally unexploited small indigenous fish species. The limitations of conventional centralized management systems highlight the importance of introducing transdisciplinary approaches which integrate limnology, fish ecology and fisheries with the interests of other resource using stakeholders and decision makers in order to develop locally appropriate co-management strategies for sustainable aquatic resource use.

**Supplementary Information:**

The online version contains supplementary material available at 10.1007/s13280-024-01996-8.

## Introduction

Tropical inland fisheries contribute significantly to improving food and nutritional security (Béné et al. 2015). Despite advances in treating inland fisheries as social-ecological systems, their value in terms of food and nutritional security is often neglected mainly due to the principal focus on marine fisheries and ocean management (Funge-Smith and Bennett 2019). Similarly, their conservation and management have been largely overlooked by policy makers. Lynch et al. (2016) and Cooke et al. (2021) emphasized the importance of inland fisheries as they make a substantial contribution to meeting food supply for billions of people worldwide. Poor and undernourished populations are particularly reliant on inland fisheries because of the critical role of inland waters in providing locally sourced, low-cost protein (McIntyre et al. 2016; FAO, Duke University and WorldFish 2023). As defined by FAO (2005) and Béné et al. (2007), small-scale fisheries are activities characterized as subsistence or artisanal. This sector often targets the supply of fish and fishery products to local and domestic markets, principally for home consumption (see also Kawarazuka and Béné 2010; see also FAO, Duke University and WorldFish. 2023).

The basis of this perspective paper are the research findings of a comprehensive study of reservoir and lake ecosystems in three Asian countries (FISHSTRAT project), using an integrative approach of land-use, limnology, fish ecology, fisheries, and their socio-economic significance, as presented in a book on “Aquatic ecosystems and development: Comparative Asian perspectives” (Schiemer et al. 2008; see also Simon and Schiemer 2015). Building on these foundations, this paper addresses lacustrine and reservoir fisheries in South and Southeast Asia as social-ecological systems, merging the experiences of our study with the latest developments in the various disciplines and published literature. Our main focus is on reservoirs which in South–East Asia are more important as fisheries resources than lakes, in contrast to tropical Africa. This synthesis underlines the requirement to introduce transdisciplinary approaches, which link limnology, fish ecology and fisheries with the interests of other resource users to develop locally appropriate co-management strategies for sustainability in aquatic resource use.

## Theoretical framework

Fisheries management must nowadays be embedded in broader management and conservation concepts (see Kolding and Van Zwieten 2006; Park et al. 2022), including co-produced co-management frameworks involving the various stakeholders engaged in and with the fisheries and fishing communities. The intensity of aquatic resource use in the tropics, e.g., for irrigation, hydropower production, fisheries, agriculture and clean drinking water supply, is particularly high, and stakeholder interests are partially competitive and differ in both implicit and explicit political power. This calls for comprehensive integrated sustainable water resource management. Ecosystem-oriented holistic approaches are therefore important for managing tropical lake and reservoir fisheries for the benefit of humanity.

**Fig. 1:**
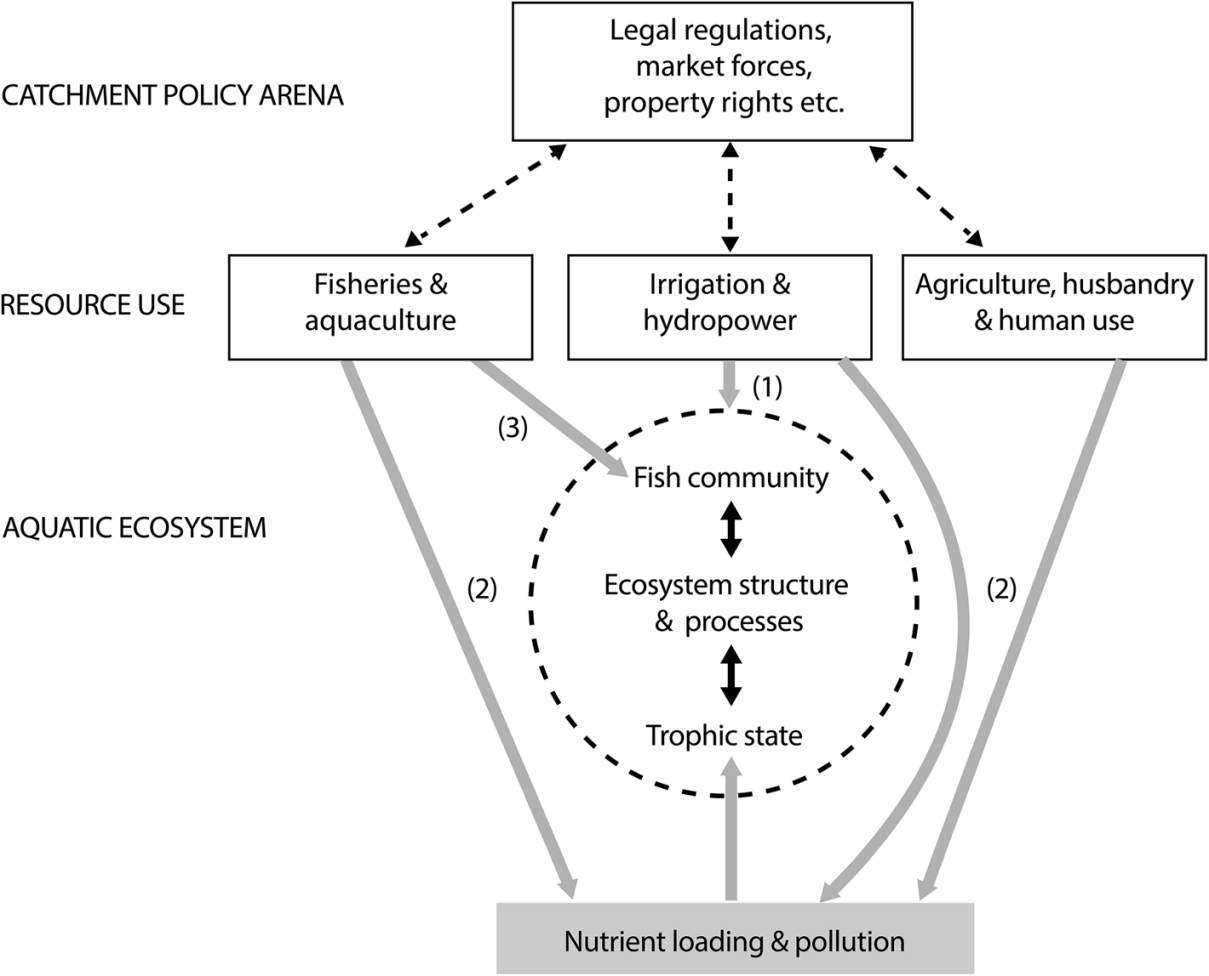
Management framework for SE Asian reservoirs and lakes. The multilevel model identifies the interactions between the “aquatic ecosystem” the “principal resource users” and the wider political–economic system (“catchment policy arena”). Reservoir ecosystems in particular, are exposed to strong pressures. The ultimate management goal in view of sustainability has to be the conservation of characteristic biota and ecosystem processes. The figure identifies 3 main types of interactions: Broken line arrows: influence by catchment policies; Black, solid line arrows: interactions within the aquatic ecosystem; Grey arrows: pressures by resource uses on ecosystem functioning; (1) effects of hydrological engineering via flushing and drawdown, (2) overloading with plant nutrients e.g. phosphorous and nitrogen e.g. by land use of the riparian zone or aquaculture, (3) effects of fisheries and overstocking, etc. (for details, see text and Fig. 2)

Figure 1 provides a conceptual framework of interactions between aquatic ecology, the manifold resource uses and the associated social-ecological system, as well as the interface between these and the policy arena. The figure sets this general framework in the context of the specific management issues of SE Asian reservoirs and lakes.

Currently most fisheries are controlled in a top–down manner, based on single-species theory, with generally poor management outcomes (Pomeroy and Berkes 1997; Puley and Charles 2022). A shift in the direction of evidence-based co-management and multi-species approaches is therefore essential. This requires a transdisciplinary approach in the form of better and more direct involvement of science and expert knowledge as well as fisher groups and communities in the decision-making process (Schiemer 2015). Within this general framework, we address the importance of aquatic studies and the potential (and risks) of two underutilized opportunities, i.e. exploitation of small indigenous fish species (SIS), and development of culture-based fisheries (CBF).

## Understanding aquatic ecosystems as a prerequisite for a sustainable fisheries development

Tropical reservoirs are highly vulnerable systems, strongly dependent on external control (land-use, human-induced hydrological regulations, water retention, water-level fluctuations) and on an array of internal regulatory processes (nutrient recycling, food chain interactions, see below). Being resources with multiple uses, inland aquatic systems are influenced by a wide range of conditions and processes from the surrounding catchment, the immediate riparian zone and within the water body itself. Since fishery dynamics are intricately connected to other supporting services of the lake/reservoir as well as to fisher communities and their social economies, it is becoming increasingly clear that all those aspects must be integrated to disentangle drivers and dynamics of change (Downing et al. 2014). Anthropogenic pressures on the reservoir and lake ecosystems result in unprecedented impact on ecosystem stability, especially causing eutrophication, which might lead to a status where the expected benefits from the ecosystems, i.e. source of water and food for people, cannot be achieved or sustained. Climate change presents an additional stressor that generally tends to enhance the trophic state and reduce the quality of water bodies (Jeppesen et al. 2012). Therefore, limnological aspects and drivers must be a central management focus.

In order to derive guidelines for sustainable resource use of these vulnerable systems, the regulatory mechanisms have to be understood. Figure 1 exemplifies in a conceptual framework, the main interactions signalling the importance of adopting a holistic approach in lake and reservoir ecosystems management. There are barriers in the interaction between the different levels which present the main challenge for an integrated planning and decision-making process (see Simon and Schiemer 2015).

The ecology of a water body is strongly dependent on its landscape setting—geology, climate, hydrology and physical structure—its morphometry, depth, shoreline development and seasonal drawdown. The hydrological regime is a key factor, especially for reservoirs, as it has significant impacts on biological productivity and fish production (Schiemer 2008; Kolding and van Zwieten 2012; Gownaris et al. 2018). Since reservoirs are constructed primarily for irrigation and hydropower generation, other—competitive—resource uses, e.g. fisheries (Amarasinghe and De Silva 2015), generally have lower priority and political influence. A principal management issue therefore relates to hydrological regulation, calling for an integrative decision-making process and the necessity to take into account the needs for drinking water supply, fisheries and the like.

A key management objective is the control of water quality and the trophic state of the water body under consideration by avoiding high loads of plant nutrients (especially phosphorus and nitrogen), organic pollutants and toxic substances and thus the threat of hypertrophic conditions, blooms of toxin-producing cyanobacteria, anoxia and subsequent fish kills. The trophic state is primarily dependent on the phytoplankton production governed by the external nutrient loading from the catchment and immediate riparian zones, as well as on internal nutrient recycling and food web processes. An array of trophic state indices has been developed mainly on the basis of data from temperate water bodies (OECD 1982). Simple limnological parameters, such as Secchi disc depth and the concentrations of total phosphorus and chlorophyll-a, and visual inspection of floating aquatic weed* (Salvinia,* water hyacinth, etc.) are extremely useful as diagnostic indicators to monitor lake and reservoir ecosystems. Most of these indices have been developed on temperate zone examples and therefore are not fully adequate for application in the tropics. Further studies are needed, specifically for the utilization in fisheries management (Cunha et al. 2021; Rast and Thornton 2005) (see below). A helpful approach is satellite based monitoring on lake levels and nutrient status, a technique which is in rapid development (e.g. Tebbs et al. 2023).

**Fig. 2:**
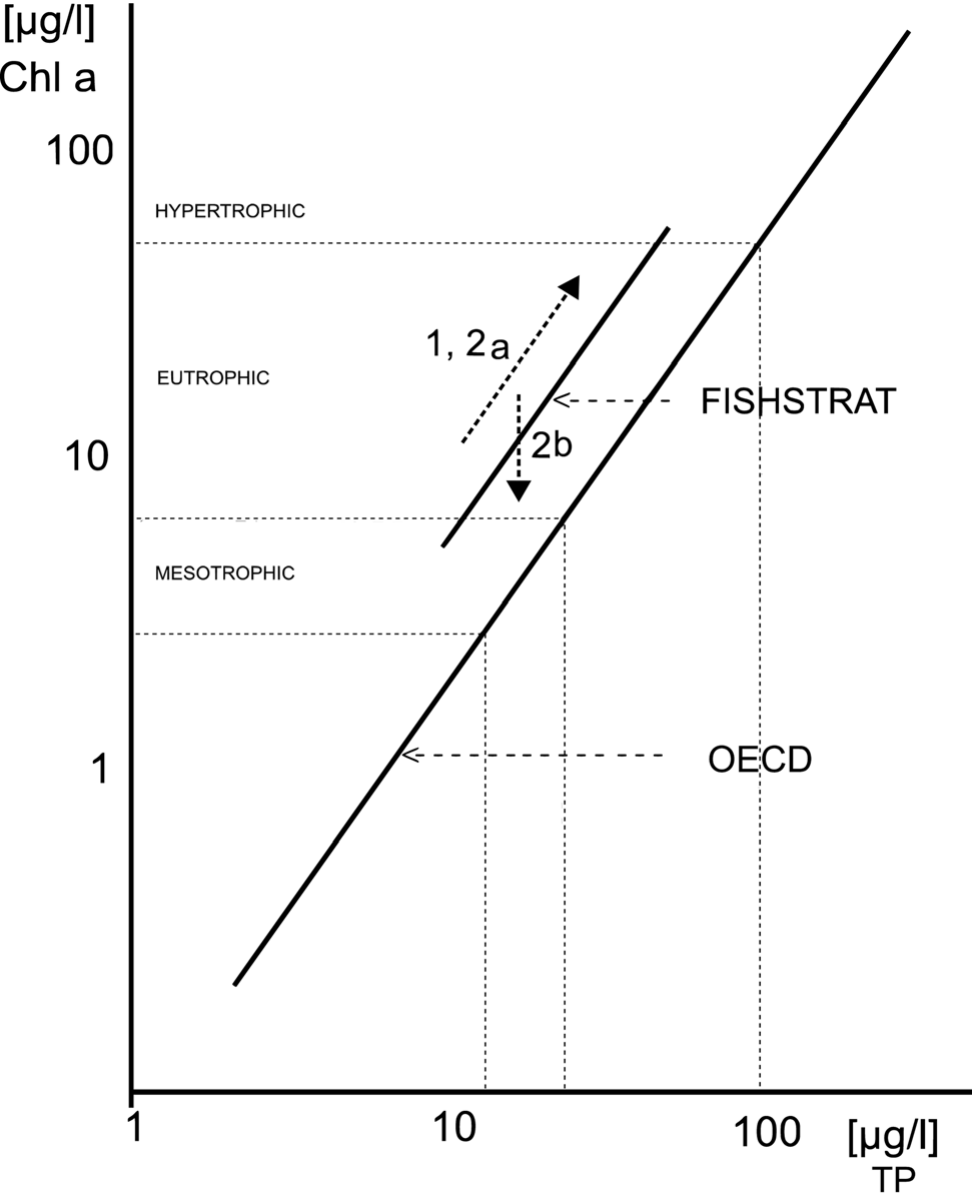
The relationship total phosphorus versus chl-a as trophic state indicator in Sri Lankan and Thai reservoirs (FISHSTRAT regression line, see Schiemer 2008) in comparison to the general regression line based mainly on water bodies of the temperate zone (OECD 1982). Our values lie within the meso- to eutrophic range of the general OECD regression line, however, exhibiting strong seasonal variation, especially in the shallow reservoirs. The figure indicates main drivers (dotted line with arrows) for this variability, namely (1) drawdown by increasing internal nutrient loading by resuspension of sediments, (2a) the internal nutrient loading by omnivorous and benthivorous fish due to bioturbation and enhancing bacterial activities at reduced water volumes of the reservoirs. These enhancing effects can be counteracted by phytoplankton grazing (2b). See text for further explanation (Schiemer 2008)

A widely used measure for defining the trophic state is the relationship between concentrations and total phosphorus (TP) and chlorophyll-a (Chl-a) in the open water column (OECD 1982; also see Abell et al. 2012; Filstrup and Downing 2017). In Fig. 2, we use this relationship, established mainly on data from temperate zone lakes and reservoirs, as a baseline for a comparison of overall results from our research on three Sri Lankan reservoirs (Minneriya, Victoria and Udawalawe) and one Thai reservoir (Ubolratana) during the FISHSTRAT project (Schiemer et al. 2008). In these reservoirs, representing a broad range of conditions in South and SE Asia, the Chl-a levels per unit TP clearly lie above the temperate zone regression lines, which means that at certain nutrient levels the phytoplankton biomass is distinctly higher. This reflects the higher phytoplankton productivity per unit biomass (e.g. Chl-a concentrations) under tropical conditions due to high temperatures and rapid nutrient recycling processes (see Talling and Lemoalle 1998; Fukushima et al. 2017; Hiroki et al. 2020). This also has been clearly exemplified by our studies on phytoplankton production in the Sri Lankan reservoirs (Silva and Schiemer 2008).

In the FISHSTRAT study, we found that the TP/Chl-a relationship exhibits high seasonal variation, especially in shallow reservoirs, induced by the hydrological regulations for irrigation and hydropower production. The increased internal loading processes at low water caused by the release of nutrients from the sediments due to wind-induced resuspension and bio-perturbation of sediments by feeding activities of omnivorous and benthivorous fish constitute major drivers of internal nutrient loading (see below) as well as the alternating drying and flooding of the aquatic terrestrial transition zone (ATTZ) which acts as a nutrient pump, (e.g. Junk 2005). In the shallow Minneriya reservoir in Sri Lanka, for example, the phosphorus levels were four times higher under low water conditions compared to higher water levels (Schiemer and Simon 2008). In contrast, seasonal changes in nutrients are less pronounced in the deep reservoirs.

### Interactions between fish and the food web

Tropical waterbodies are generally considered to be more efficient in primary production on a given nutrient basis than temperate lakes (Lewis 1996, see also Kolding and van Zwieten 2006). Besides phytoplankton production, other carbon sources supply the aquatic food webs and thus drive the productivity of fish. Macrophytes can play a substantial role both as carbon source especially via detritus formation (see below), as well as habitat structure and shelter for biota. However, in reservoirs used for irrigation or hydropower production, which experience substantial water-level fluctuations and seasonal drawdowns, intensive growth of macrophytes is largely precluded. Floating weeds are an exception since they are much more resilient to water-level fluctuations.

Riparian transition zones provide conditions for intensive terrestrial plant growth and its decomposition after flooding as well as grazing by terrestrial herbivores and subsequent dung deposition. This contributes significantly to the aquatic carbon cycle in shallow reservoirs, similar to what happens in floodplain rivers (Junk 2005; Mosepele et al. 2022). Thus, reservoir ecosystems are often in a heterotrophic state driven by allochthonous material and microbial processes (Peduzzi and Schiemer 2008). Tropical zooplankton species are generally of smaller size than in temperate zones (Gillooly and Dodson 2000) (see below) indicating the generally higher turnover rates under tropical conditions and a higher predation pressure by fish. The same pattern is found for the generally small-sized soft-bottom zoobenthic community, indicating that macro-zoobenthos represents a main energy pathway to fish (Schiemer 2008).

**Fig. 3:**
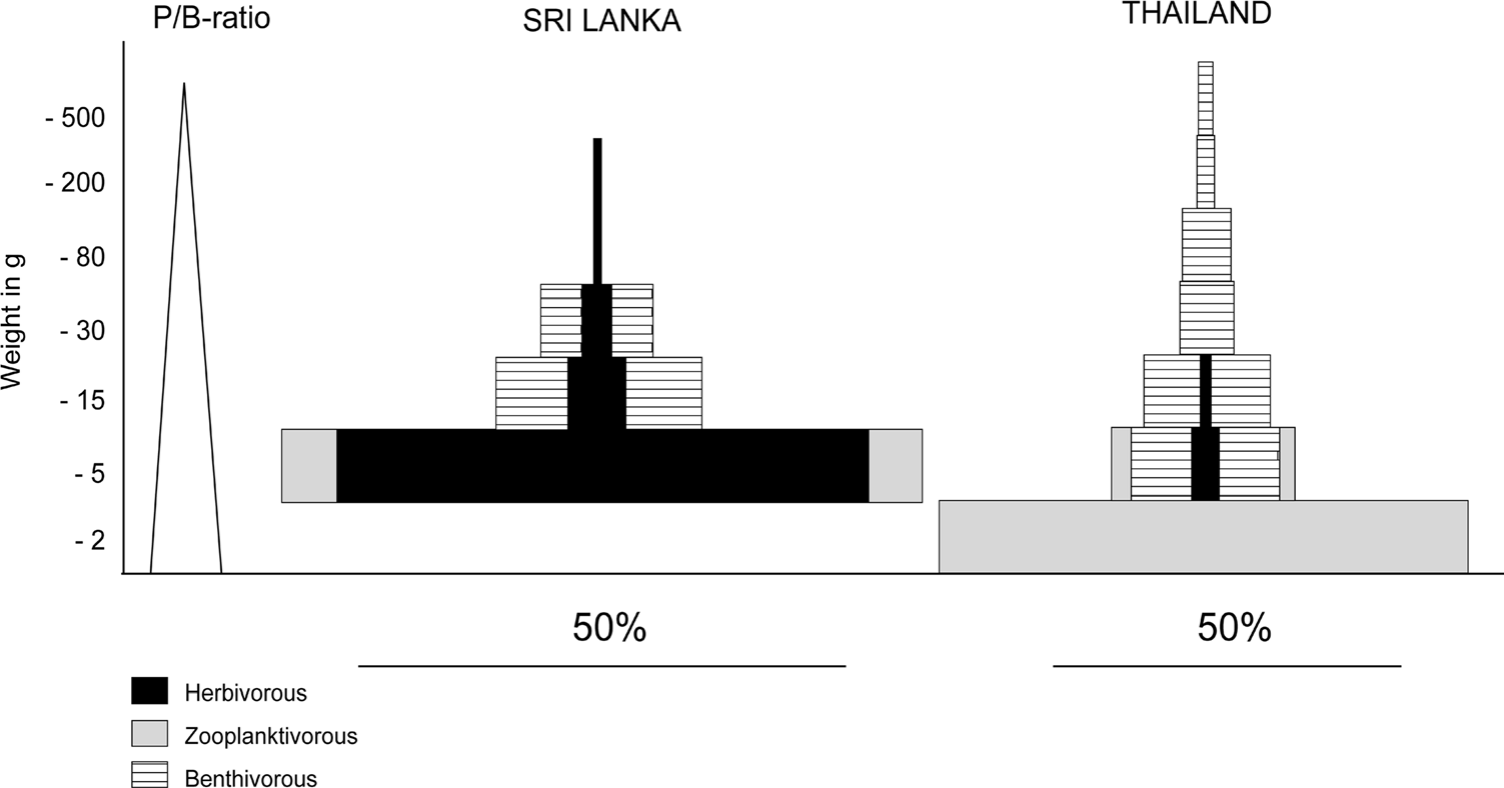
Comparison of biomass (in percentages of total fish biomass) versus size classes of fish in Sri Lankan and Thai (Ubolratana) reservoirs based on a combination of hydroacoustic surveys and multi-mesh gill net analysis. The trophic structure is indicated. The group labelled as “herbivorous” includes small-sized seston-filtering species and “tilapias” which essentially are omnivorous. However, their digestive physiology indicates a high significance of plant material in their diet (Hofer and Schiemer 1981, 1983). The annual production/biomass ratio (*P*/*B*) is declining with the size of fish, as schematically indicated. This inverse relationship between size and turnover rate (*P*/*B*) is well documented (e.g. Banse and Mosher 1980) (from Schiemer 2008)

Ideally, a prerequisite for optimizing fisheries offtake is a detailed quantitative assessment of the fish community of a particular water body. In practice, this will be impossible for smaller waterbodies as the data requirements would be more expensive than the value of the fishery and simpler methods have to be applied (see Kolding and van Zwieten 2014). The challenge is to understand the habitat conditions and habitat utilisation of the different guilds, the food base and carrying capacity of the native fish community versus introduced exotics like *Oreochromis* spp. and possible top–down effects via food chain cascades. Quantitative information of this kind is also required for a meaningful application of mass balance models, e.g. the Ecopath software (see Christensen and Pauly 1993; Kolding et al. 2016), to elucidate the trophic functioning of a particular lake or reservoir ecosystem. A detailed understanding of the fish stocks and their dynamics requires a combination of conventional methods of experimental fishing and the application of scientific hydroacoustics (Tessier et al. 2016). Hydroacoustics are a powerful tool to gain insight into the size and biomass structure of fish populations, although their value is limited under certain conditions, e.g. in littoral areas (Prchalová et al. 2003). During the FISHSTRAT project, the combination of investigations included multi-mesh gillnetting, shore seining for small pelagic fish, analysis of fisheries catches (Sricharoendham et al. 2008), the use of scientific hydroacoustics (Prchalová et al. 2003) and a detailed analysis of feeding patterns (Amarasinghe et al. 2008; Ariyaratne et al. 2008). The multi-species assemblages differ greatly between a diminished island fauna in Sri Lanka, the species-rich assemblages of Ubolratana reservoir in Thailand, and the deep Lake Taal in the Philippines. The different assemblages exhibit distinct spatiotemporal patterns and complex food web relationships (see Schiemer 2008). Notwithstanding all these differences, however, we found one significant common characteristic in the different water bodies studied, namely the presence of high densities of small-sized fish, which are highly productive, but are generally overlooked by traditional fishing methods (Fig. 3). This confirms earlier records on biomass dominance by small-sized species (Schiemer and Hofer 1983). These species represent an enormous resource underutilized by traditional fisheries (see below).

In all the Sri Lankan reservoirs studied, *Amblypharyngodon melettinus,* a seston-filtering cyprinid feeding with a remarkable diurnal cycle in migration and filter-feeding (Hofer et al. 2003), shows a dominance of over 50% of the biomass and even greater proportion of biological productivity (Pet et al. 1996). Ubolratana reservoir is dominated numerically by the zooplanktivorous Thai river sprat (*Clupeichthyes aesarnensis*), a minute gobiid *Gobiopterus chuno* and many small-sized cyprinids (see Schiemer 2008).

### Trophic interactions, top–down control and the potential for biomanipulation

Carpenter and Kitchell (1993) introduced the “trophic cascade concept” for temperate lakes, which highlighted that the components of higher trophic levels in the food web can control lower trophic levels and determine system processes and trophic states. Such top–down mechanisms have been successfully applied in temperate zone water bodies to improve the trophic state by controlling zooplanktivorous fish and thus allowing large-sized zooplankton species with high filtering capacity (e.g. *Daphnia*) to develop.

However, this temperate zone model of biomanipulation cannot readily be transferred to tropical reservoir conditions because of striking differences in the structure of the fish communities, e.g. higher species richness, widespread omnivory, lower densities of strict piscivores and a generally higher fish biomass (Jeppesen et al. 2012). The food chain effects, i.e. the shaping of the biocoenotic structure by grazing and predation, are less well understood in tropical aquatic ecosystems than in temperate regions (see Gownaris et al. 2018). The regulatory effects of fish–zooplankton interactions on phytoplankton appear to be less significant in the tropics than in the temperate zone.

Our data (Schiemer 2008; Schiemer and Simon 2008) provide evidence that omnivorous “tilapias” (especially *Oreochromis mossambicus* and O. *niloticus*) can significantly affect the trophic state of a water body (see Fig. 2). At high population densities of younger stages in the filter-feeding mode, they can reduce phytoplankton biomass by grazing (Fig. 2: arrow 2b) (see also Schiemer 1996). More importantly, however, the larger omnivorous size classes of *O. mossambicus* and *O. niloticus*, which use a benthic feeding mode, have a stimulating eutrophication effect (Fig. 2: arrow 2a) by enhancing nutrient release from the bottom strata through faeces production and enhanced bacterial activity. Small-sized cyprinid species like *A. melettinus* tend to shift the phytoplankton community to cyanobacteria and, in turn, stimulate primary production and eutrophication (Hofer et al. 2003).

**Fig. 4:**
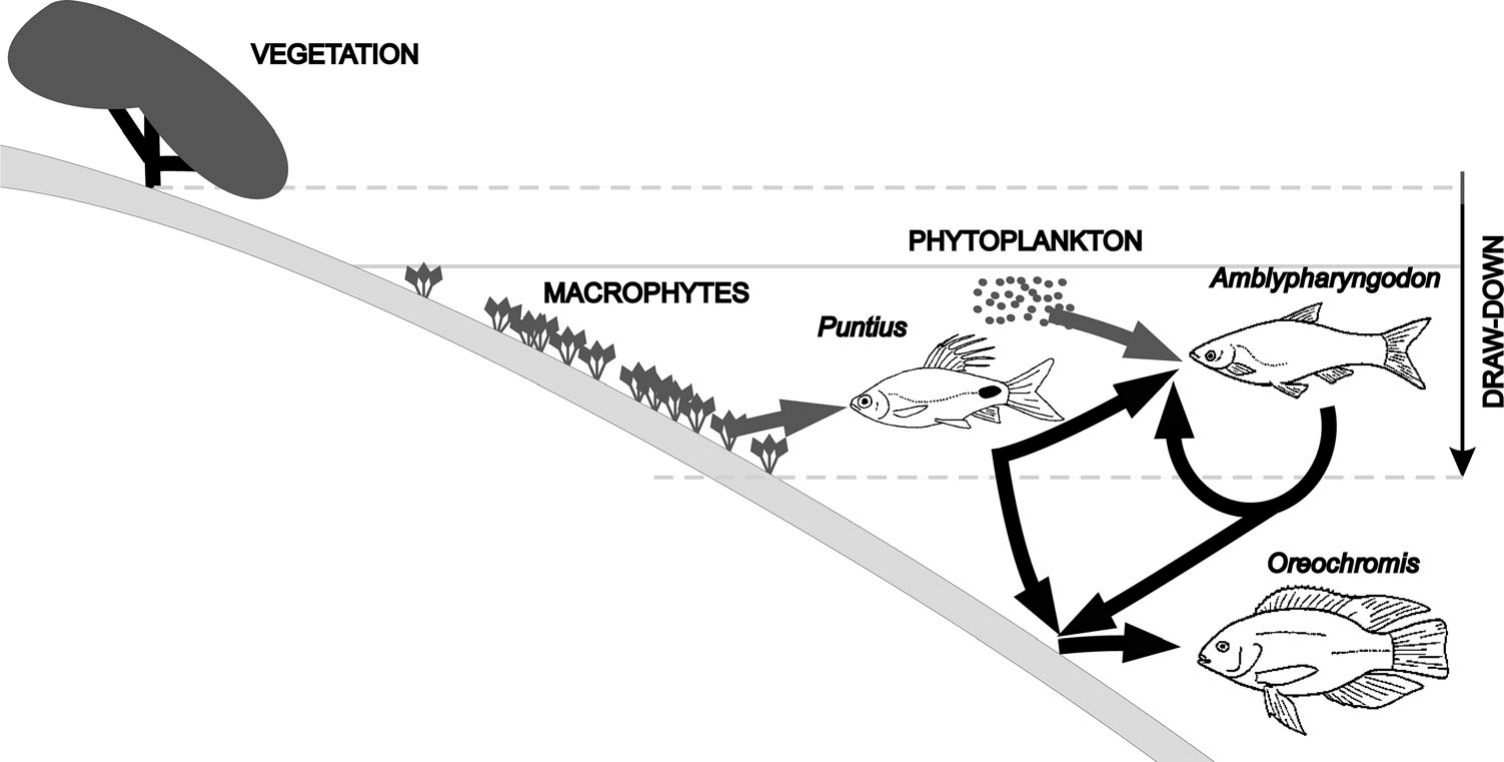
Major carbon pathways of “herbivorous” fish (see also Fig. 3) in Sri Lankan reservoirs. Two primary autochthonous carbon sources are identified: phytoplankton production and littoral growth (semi-terrestrial and aquatic macrophytes and periphyton growing upon them). A large part of the carbon (and energy flow) combined with microbial nutrient recycling processes occurs via detritus. Littoral exposure due to drawdown enhances terrestrial/semi-terrestrial macrophyte growth, which in turn decompose during inundation (from Schiemer 2008)

A coherent understanding of biotic interactions and their significance for trophic state conditions in tropical lakes and reservoirs is therefore important. High population densities of omnivorous fish and the worldwide introduction of “tilapias”, in particular, call for a detailed analysis of their functional role (see Figs. 2 and 4).

Figure 4 illustrates the complex interactions of herbivorous/omnivorous fish in shallow Sri Lankan reservoirs. Apparently, these complexities restrict the applicability of simple biomanipulation measures by the population control of such species.

### HOW TO INCREASE FISHERY YIELDS AND IMPROVE FOOD SECURITY WHILE APPLYING SUSTAINABLE RESOURCE MANAGEMENT

Although the growth of aquaculture has during the past three decades contributed to narrowing the gap between supply and demand for food fish, even at the current rate of fish consumption, the world will require an additional 30–40 million tonnes by 2050 (De Silva 2016). Intensification of aquaculture in the form of cage culture in tropical lake and reservoir ecosystems is a mixed blessing, unarguably leading to serious damage to the environment when not carefully controlled. There are many records of negative effects of intensive cage culture both from Asia and Africa due to eutrophication effects (e.g. Abery et al. 2005; Aypa et al. 2008) as well as escape of invasive species and diseases (tilapia lake virus, for example). Importantly, these events impacted both wild and cage fish populations. Furthermore, climate change impacts are likely to worsen these issues (De Silva and Soto 2009). Cage culture requires 2–5 times more fish protein, in the form of fish meal, to feed the farmed species than is supplied by the farmed product and is the source of much water pollution. Fish in cages needs to be fed intensively, and the feed needs high concentrations of expensive protein and fat. Actually, cage culture produces less protein than it consumes, which needs to be sourced somewhere (see Tacon and Metian 2015). It is therefore imperative that, instead of aquaculture intensification, other potential means of increasing food fish production should be considered for improved food security.

Capture fisheries yields in Asian lakes and reservoirs reveal remarkable diversity due to differences in the limnology of individual water bodies, their trophic state, size, basin type and hydrology as well as the intensity of local fisheries and the selective exploitation of the available fish biomass (e.g. van Densen and Morris 1999). In many Asian countries where productive inland fisheries exist, such as Indonesia, the Philippines and Sri Lanka, introduced exotic cichlids and Chinese and Indian major carps produce high yields (Amarasinghe and De Silva 2015). The unprecedented success of introduction of exotics in many tropical reservoirs is due to their ability to occupy “vacant niches” in reservoir ecosystems and their hardiness to survive in eutrophic waterbodies (De Silva et al. 2009). Previously it was generally believed that tropical reservoirs lacked indigenous lacustrine fish species (e.g. Fernando and Holčik 1991). It is now well established that inland water bodies produce sizeable populations of small indigenous fish species (SIS) including small pelagics, which often remain unexploited or underexploited, indicating a high potential for increasing fish production in reservoir ecosystems (Amarasinghe et al. 2016; Kolding et al. 2019, 2023) (see below).

### Strategies for more complete utilization of biological productivity

Exploitation patterns across trophic levels in aquatic food webs are highly unbalanced, as shown for Asian reservoir fisheries on the basis of mass-balanced models (e.g. Villanueva et al. 2008; Panikkar et al. 2021) and also for 110 different ecosystems worldwide (Kolding et al. 2016). In accordance with the ecosystem approach to fisheries, a “balanced harvest” concept (BH) has been proposed, i.e. harvesting all components in the ecosystem in proportion to their productivity (Garcia et al. 2012; Law et al. 2012). Plank et al. (2017) have shown that small-scale gillnet fishers, operating without size-based regulations, would end up catching small and large fish in proportion to their productivity if the market is not distorted by consumer preferences. They emphasized small-scale fisheries in areas where poverty and malnutrition are critical, and provision of biomass for food is more important than the export market value of the fish harvest. In this perception, the aim is a more complete exploitation of the biological productivity across the food webs and not harvesting just a few table-sized fish. Conventional single-species fisheries management approaches, such as surplus yield models and dynamic pool models, which essentially aim at controlling the extent of fishing, are not adequate for effective multi-species management (Kolding and van Zwieten 2011; Kolding et al. 2023).

### Exploitation of small fish

In Asian reservoirs, the natural fish stock consists of high densities of “small fish”, according to the FAO definition of “small fish”, namely all fish smaller than 25 cm in length (Bavink et al. 2023). This group of fish includes the highly diverse group of small indigenous fish species (SIS) and small pelagics. Most species feed at lower trophic levels—at the bottom of the food chain (Kolding et al. 2023). Their production per unit biomass (*P*/*B* ratio, see Fig. 3) is high. Therefore, from the first principles of environmental energy flow, targeting small fish is an efficient utilization of available food production and a strategy to optimize fish yields (Kolding et al. 2023).

Unlike in tropical Africa, Asia reports only a small contribution of small fish in the total catch (mainly because of lack of reporting). However, there is an increasing trend for a subsidiary SIS fishery. Despite increasing evidence of high fishery potential of SIS in Asian reservoirs (Pet et al. 1996; Amarasinghe et al. 2016, see below), this nutritionally rich resources remain widely underexploited or unexploited. Examples from Sri Lanka, Thailand and Laos show the potential for productive subsidiary fisheries on small fish (Mattson et al. 2001; Athukorala and Amarasinghe 2020; Amarasinghe and De Silva 2015). A SIS fishery is also reported from small waterbodies in Bangladesh (Islam et al. 2023). In Sri Lanka, previous studies have shown that SIS could be differentially exploited using gillnets of stretched mesh sizes, 15–38 mm without adverse effects on the fishery of larger commercially important fish species such as *Oreochromis mossambicus* and *O. niloticus* (e.g. Pet et al. 1996; Amarasinghe and De Silva 2015), and on the stocked carp fingerlings for culture-based fisheries (CBF) development (Athukorala and Amarasinghe 2020). Such a strategy for SIS exploitation without adverse impact on existing commercially important fish species is possible since they occupy different micro-habitats: juvenile tilapias and stocked carp fingerlings that are susceptible to small-mesh gillnets occupy littoral areas, whereas SIS occupy limnetic areas (Amarasinghe et al. 2016). The reasons for the present under-exploitation of SIS and small pelagics are regulatory constraints, such as gillnet mesh size limits (Sugunan 2010; Amarasinghe and De Silva 2015) and lower consumer preferences compared to table-sized tilapias (Amarasinghe et al. 2016).

The nutritional contribution of small fish and their efficacy in reducing or preventing malnutrition in developing countries are comprehensively reviewed by Thilsted (2012) and Bavinck et al. (2023). Such a fishery could contribute to an increase in fish yields and provide cheap, high-quality human food contributing to the nutritional security of rural communities. Aura et al. (2022) examined existing policy and institutional documents on fisheries, health and trade to assess the level of inclusion of small indigenous fish species. They found that small fishery resources are underutilized and there is a general need for a targeted approach to realize its potential for nutritional security. We therefore recommend that the relevant fisheries authorities take steps to legalize and encourage sustainable exploitation of small fish in Asian tropical lakes and reservoirs. The exploitation of small fish is an additional means of increasing fish production through a more complete utilization of biological productivity in lacustrine ecosystems. At present, the question of the contribution of small fish in the total inland fisheries catches is difficult to answer because their catch is underreported, especially in countries where fishing on small size classes is officially prohibited (FAO, Duke University and WorldFish 2023). More research and monitoring are urgently required before we can understand the full potential of small fish in the aquatic ecosystems of tropical Asia without depleting recruitment of large species and thereby undermining fisheries that depend on them (see supplementary files).

### Culture-based fisheries

Culture-based fisheries (CBF), a strategy for better utilization of biological productivity in reservoir ecosystems, can be a way forward for inland fisheries development in most Asian countries (De Silva 2003).

Omnivorous tilapia and exotic carp species contribute significantly to inland aquaculture and fisheries enhancement (De Silva et al. 2004). It has been reported that stocking of these non-native fish species in substantial numbers in Asian inland waters did not have adverse impact on the native fish biomass (Arthur et al. 2010). In CBF, hatchery-reared fish fingerlings of different fish species are stocked for subsequent recapture, which is analogous to “polyculture”. The large number of irrigation reservoirs in the Asian region can be utilized in this respect. As many tropical countries, including Sri Lanka, do not have native fish species which grow fast to a relatively large size, fast-growing exotic species such as Chinese and Indian major carps are essential for the CBF development. Remarkable successes have been achieved in CBF development in Sri Lanka, Vietnam and Lao PDR (Amarasinghe and Nguyen 2010; Phomsouvanh et al. 2015). In CBF, unlike in conventional aquaculture, external inputs, such as feed, are not involved, which makes this strategy environmentally less disturbing compared to conventional aquaculture practices (De Silva 2003).

CBF strategies in reservoirs are essentially communal activities, and the fish stocks are generally owned and managed either individually or collectively. CBF have a strong social component that is of crucial importance to their success. A recent study evaluating strategies adopted by fisher communities for CBF development in five Sri Lankan irrigation reservoirs (Pushpalatha et al. 2020) showed that institutional robustness in the fisher communities for making management decisions, as spelled out by Cox et al. (2010), increased the mean annual income of fishers. Leadership qualities, empowering fishers for management decision-making, and the ability to work with others (horizontal integration) are shown to be important factors for the success of CBF.

From the point of view of sustainable resource management, it is necessary that measures to improve fisheries output have to be carefully evaluated, balancing the benefits and the potential adverse effects and hazards for ecosystem health and local biodiversity (e.g. De Silva and Funge-Smith 2005; De Silva et al. 2009; Pauly et al. 2016). Detailed interdisciplinary studies on the effects of fisheries management practices on the aquatic ecosystems and their biota are a prerequisite for further improvements.

## The potential of co-management frameworks and the challenge of transdisciplinarity

Following from the previous point, it is now well established that traditional, top–down management by centralized governance authorities is inappropriate (Andrew et al. 2007) and has often failed in the past because of inadequate consultation of and participation by fishers (Simon and Schiemer 2015; Schiemer and Simon 2008). They feel ignored or even alienated and lack an adequate sense of shared ownership of the resources. Hence, they often lack confidence in the basis for maximum sustainable yield quotas in capture fisheries, minimum gillnet mesh sizes or maximum cage cover limits in aquaculture, which are often also not equitably distributed within and between communities and may exclude others altogether. Under such circumstances, fishers have little incentive to adhere to quotas and other regulations with which they do not agree (see Kolding and van Zwieten 2011; Plank et al. 2017).

The challenge is how to get the fishers to arrive at some level of consensus internally and with other stakeholders involved. Successful sustainable resource use requires co-management involving the different stakeholders based on solid empirical, scientifically sound data in combination with local knowledge and traditions. The main challenge is that a system-understanding of the resource base should be accepted by all resource users. Fishers and other stakeholders should participate actively in generating these data and understand and negotiate the consequences of their resource use (see, for example, Ticheler et al. 1998). This has generally proved impossible under conventional regimes and practices, and the transdisciplinary co-design/co-production approaches examined here appear to offer greater potential for achieving progress.

Conventional fisheries management regulations are generally based on single-species theories and approaches focusing on maximizing production and revenues from single-species populations. These principles, developed in industrial marine fish resource utilization, are largely inapplicable in small-scale multi-species fisheries. The theoretical foundation for the conventional single-species legislation in a multi-species framework is increasingly being challenged, indicating the need for a fundamental change in the management philosophy, e.g. a re-orientation from single-species to multi-species theory (Kolding and van Zwieten 2011; Zhou et al. 2019). There is significant potential for increased production and more balanced exploitation if the overall fishing pressure were directed away from large fish towards small fish also in Asian contexts (see above). Trends in this direction are ongoing but often take place without scientific evaluation of pressures, ecosystem effects or governance, and often conflict with current regulations in many regions (Kolding et al. 2023). Moreover, regular review is required to ensure that rules and procedures are updated to become or remain equitable and sustainable, particularly in the context of changing conditions.

Transdisciplinarity, the building of a science-policy bridge, requires the crossing of boundaries separating various academic disciplines such as limnology, fisheries and socio-economy from various fisher stakeholder interests, professional regulatory and management issues, and political decision processes. This approach demands a paradigm shift of thinking from conventional “experts know best” assumptions, to “resource-users know as much”. This would enable the conflicting rationalities of different stakeholders—based on their respective training and previous experience (and hence origins and history of the particular resource conflict)—to be understood and accommodated, as has been demonstrated in other multi-stakeholder contexts (Watson 2014; Smit et al. 2021). The diversity of stakeholders involved usually makes attaining social sustainability a slow process, requiring complex negotiations in search of consensus or at least an adequate basis for majority decision-making. Although a vital prerequisite for maximizing prospects for eventual success and sustainability, the time and uncertainty involved in such processes often lead politicians and civil servants to avoid them and to rely on inadequate short-cut decisions or on conventional regulations (Simon 2021; Puley and Charles 2022).

Conventional wisdom previously held that, in the absence of centralized management in many fisheries, self-interest prevails over collective welfare, resulting in resource depletion and degradation, so-called tragedy of the commons in relation to so-called open-access resources (Hardin 1968). However, reality is more complex. Many such resources are more accurately understood as common-pool resources (Ostrom et al. 1994; Ostrom 2008; Kolding and van Zwieten 2011; Haikkila and Carter 2017), because they are not accessible (both physically and in terms of rights) to literally anyone but only to members of particular communities and user groups. Indeed, communities and user groups often initiate their own regulatory systems, drawing on local indigenous knowledge and culture that have evolved over time (Amarasinghe and De Silva 1999; Bavinck 2005; Schiemer and Simon 2008). This situation, where customary rules and regulations apply to fisheries management in the absence of or in addition to state law, can be seen as legal pluralism, defined as a situation where different legal principles and systems are applied simultaneously (Jentoft et al. 2009). These situations can be the cause of conflicts in small-scale fisheries (Kolding and van Zwieten 2014) and, indeed, often lead to destructive situations.

Such conflicts reflect unilateral and non-consensual actions by different parties. Particularly when the stakeholder groups involved have very different capacities, the asymmetrical power relations among them are problematic but need to be addressed, often through involvement of independent facilitators or mediators. Prospects for avoiding conflicts and promoting sustainability are maximized when the various stakeholders engage positively with one another through consultative processes in which all forms of knowledge and experience—not just scientific or “expert” knowledge—are respected and considered. Our earlier study already identified many of the potentials and constraints to developing transdisciplinary co-management regimes in the different contexts of fisheries in Sri Lanka, Thailand and the Philippines. We subsequently updated the analysis to take account of the expanding literature and its greater emphasis on cultural and governance dimensions in ever more contexts (Simon and Schiemer 2015).

Co-management regimes and strategies formulated and implemented according to the above principles—including mediating asymmetrical power relations—and adapted to the particular situation in each locality offer the most meaningful way to empower fisher communities and ensure equitable resource access for different community and livelihood groups. Where capture- and culture-based fisheries co-exist and may have competing interests, the more complex situation may require different regimes from situations where this does not apply. This also generally requires governmental intervention (Pomeroy and Berkes 1997). Amarasinghe and Nguyen (2010) and De Silva (2016) also highlighted the often-communal nature of CBF in small reservoirs as lending themselves to co-management.

The importance of local appropriateness must be emphasized because situations can vary, even between neighbouring communities. For example, in some cases, the same people may practice both capture fisheries and cage aquaculture but elsewhere these activities are undertaken by different people. Particularly in large water bodies, such as Ubolratana reservoir in Thailand and Lake Taal in the Philippines, the socio-economic diversity of littoral communities can be substantial, while commercial-scale cage owners may be wealthy outsiders using local community members as operators (Aypa et al. 2008; Schiemer and Simon 2008; Simon et al. 2008).

Puley and Charles (2022) distinguish six elements of fishery co-management activities, namely direction-setting, planning and policy development; harvest management; compliance and enforcement; ecosystem stewardship, conservation, rehabilitation; research; and organizational management and development. These place different demands on the various stakeholders and will affect their willingness to undertake them. The great diversity of situations worldwide involves different combinations of these elements as well as relative (im-)balances of power relations among the stakeholders. This underlines the importance of identifying locally appropriate forms of co-management regimes and arrangements, in accordance with the principles and guidelines elaborated in the recent IPBES report on the sustainable use of wild species (Park et al. 2022). In practice, this may impose constraints on the viability and sustainability of such management systems, particularly over the longer term, if the time commitments and burden of responsibilities are substantial and perceived as being shared inequitably (see also Hemström et al. 2021; Puley and Charles 2022). Furthermore, particularly under conditions of accelerating environmental change, fishery situations are dynamic rather than static. Hence, ongoing monitoring and periodic review are necessary to ensure that rules, catch limits and other parameters remain sustainable and appropriate. While therefore clearly neither a sinecure nor a guarantee of success and sustainability, co-produced co-management systems maximise the potential for addressing conflicting rationalities and interests constructively and promoting resource and livelihood sustainability.

## Summary, conclusions and recommendations

Tropical lakes and reservoirs, particularly in Asia and Africa, support small-scale fisheries. They contribute significantly to the food and nutritional security of local people. It is therefore asserted that there should be greater focus in national strategies on sustainable management of this valuable resource base. A synoptic approach to fisheries management of tropical lakes and reservoirs and their riparian environment is required as the basis for co-designed and sustainable compromises among the interests of diverse users of diverse political power and strength. It is suggested that management should be based on an interdisciplinary combination of analysis of the sensitivity and reactivity of the freshwater resource, on socioeconomics of its resource-use, a cross-sectoral engagement with other water users and a transdisciplinary approach combining research-based evidence with a co-designed decision-making process.

Limnological studies provide an important basis for inland fisheries management. Particularly relevant are aspects of water quality control, the understanding of food web dynamics, and the understanding of the habitat use and population dynamics of key species in the fish communities, which is completely lacking in conventional single-species theory and subsequent management recommendations. In combination with fisheries research, the application of multi-species population models (e.g. Ecopath) as well as stock assessments using a combination of conventional methods and hydroacoustics, such studies will allow the exploration and exploitation of “untapped resources” (e.g. SIS). Unexploited fishery resources can be used for human or animal food. Manipulation of fish communities in reservoirs and lakes, e.g. introduction of exotic species at low trophic levels, has proven useful for effective utilization of trophic resources such as the introduction of the small zooplanktivore sardine (*Limnothrissa miodon*) in several African lakes and reservoirs (Kolding et al 2019). CBF is a fisheries enhancement strategy that has many benefits for local populations. The combination of limnological and fisheries studies allows the assessment of the potential of profitable enhanced fisheries (e.g. CBF) to be introduced. It also provides the basis for an evaluation of the potential and hazards (water quality) of aquaculture as, for instance, cage culture.

We suggest that successful sustainable resource use requires co-management involving the different stakeholders based on solid empirical, scientifically sound data, including indigenous local knowledge and mediation of unbalanced power relations. Fishers and other stakeholders should participate in negotiating management plans. The diversity of stakeholders involved usually makes attaining social sustainability a slow process, requiring complex negotiations in search of consensus or at least an adequate basis for majority decision-making. External professional facilitation can be helpful in managing power asymmetries and overcoming pre-existing antagonisms, especially during the early stages.

Co-management strategies should be adapted to each local situation but offer the most meaningful way to empower fisher communities and ensure equitable resource access for different communities and livelihood groups when endowed with effective voice through participatory power in decision-making (Hemström et al. 2021; FAO, Duke University and WorldFish 2023).

It is a fact that the communal nature of CBF in small reservoirs often favours the introduction of co-management. The importance of local appropriateness must be emphasized because situations can vary, even between neighbouring communities. Particularly in large water bodies, the socioal-economic diversity of littoral communities can be substantial. While therefore clearly not a sinecure, co-management systems maximize the potential for addressing conflicting rationalities and interests constructively and promoting resource and livelihood sustainability. In most cases, this will require the involvement of local or higher authorities.

Since tropical lake and reservoir fisheries are mostly rural economic activities, although often directly connected to urban and regional markets for their products and for commercial inputs, it is imperative for their sustainable management that both the resource component itself and the social component which makes use of the resource should be given appropriate weight in management planning.

An inherent characteristic of lake and reservoir fisheries is that several stakeholders are involved in resource utilization, hence requiring their consent and collaboration (i.e. transdisciplinarity). As detailed above, there is a need for a paradigm shift from the conventional “expert-centred” approach to understand and accommodate the often-conflicting rationalities of different stakeholders, including fisher communities, in the management decision-making process but active and dedicated contributions by fisheries authorities for social mobilization remains essential.

### Supplementary Information

Below is the link to the electronic supplementary material.

Supplementary file1 (PDF 7309 KB)
