# Supplementary material for: Sustainable aquatic resource management and inland fisheries in tropical Asia: Interdisciplinary and transdisciplinary approaches
Source: Ambio. 2024 Mar 18;53(7):1050–64. doi: 10.1007/s13280-024-01996-8 (PMC11101390; doi:10.1007/s13280-024-01996-8)

Ambio

Electronic Supplementary Material

Title: **Sustainable aquatic resource management and inland fisheries in tropical Asia: interdisciplinary and transdisciplinary approaches**

Authors: Fritz Schiemer, Upali S. Amarasinghe, David Simon, Jacobus Vijverberg

Photo 1: Gill-net fisherman at Senanayake Samudra, Sri Lanka. (Photo U.S.Amarasinghe)

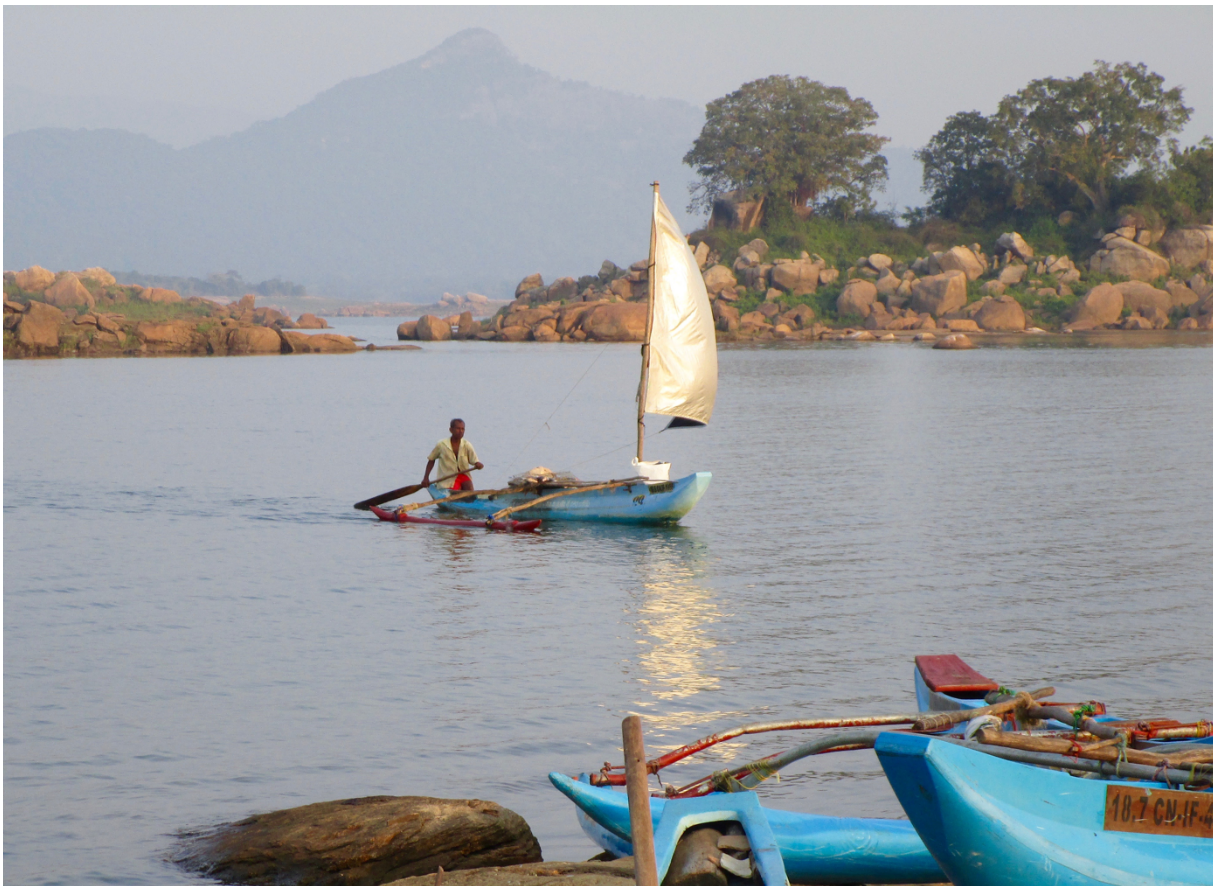

Photo 2: Cast-net fishermen at Ubolratana reservoir, Thailand. (Photo S. Ingthamjitr)

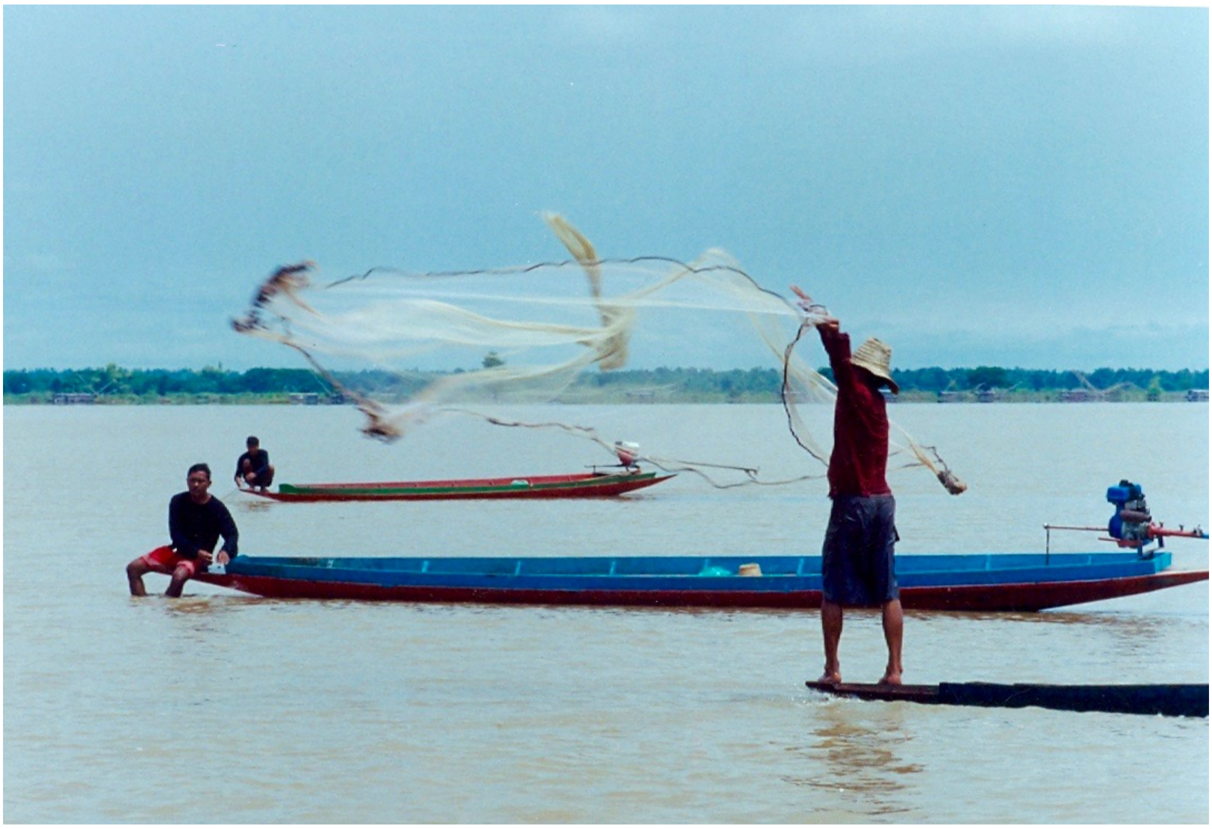

Photo 3: Fish drying of halfbeak, *Hyporhamphus limbatus*, at Minneriya reservoir, Sri Lanka. (Photo F. Schiemer)

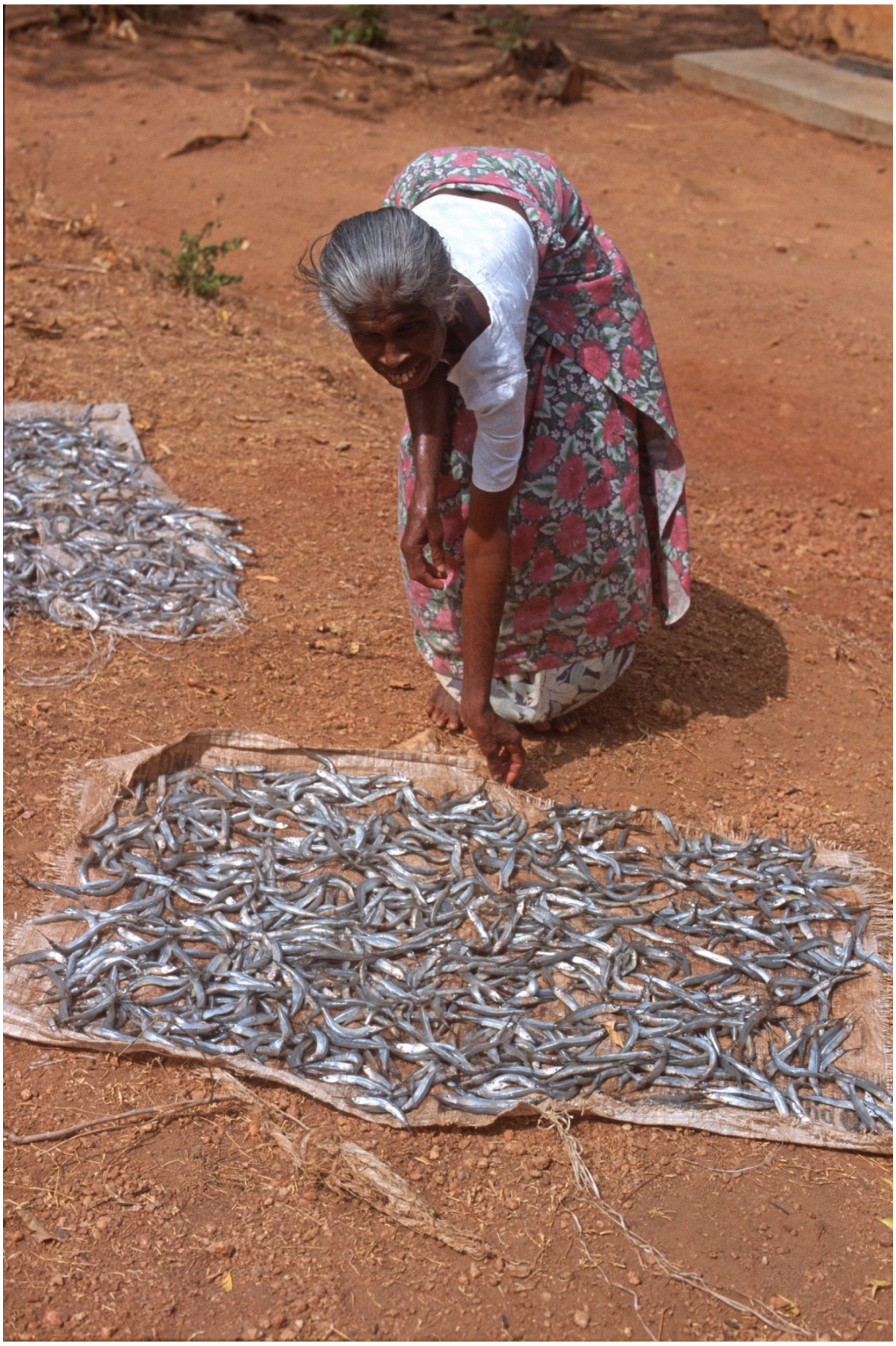

Poto 4: Small-sized fish being processed at Ubolratana reservoir, Thailand. (Photo D. Simon)

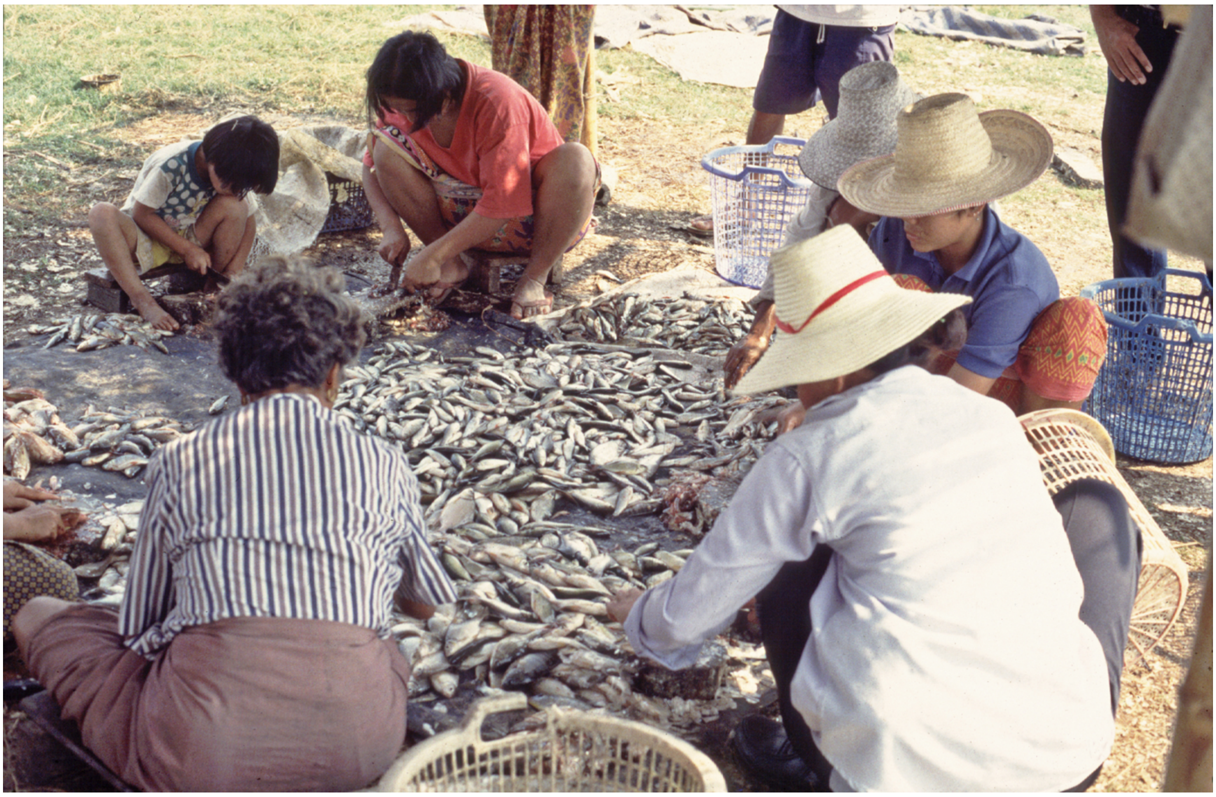

Supplement: Supplementary file 1 — Supplementary file1 (PDF 7309 KB) [file 13280_2024_1996_MOESM1_ESM.pdf]
